# Cell senescence in neuropathology: A focus on neurodegeneration and tumours

**DOI:** 10.1111/nan.12689

**Published:** 2021-02-01

**Authors:** Gabriela Carreno, Romain Guiho, Juan Pedro Martinez‐Barbera

**Affiliations:** ^1^ Developmental Biology and Cancer Programme Birth Defects Research Centre Institute of Child Health Great Ormond Street Hospital University College London 30 Guilford Street London WC1N 1EH UK

**Keywords:** Alzheimer's disease, cell senescence, craniopharyngioma, diffuse midline glioma, glioblastoma multiforme, low‐grade glioma, medulloblastoma, multiple sclerosis, Parkinson's disease, SASP, senolytic

## Abstract

The study of cell senescence is a burgeoning field. Senescent cells can modify the cellular microenvironment through the secretion of a plethora of biologically active products referred to as the senescence‐associated secretory phenotype (SASP). The consequences of these paracrine signals can be either beneficial for tissue homeostasis, if senescent cells are properly cleared and SASP activation is transient, or result in organ dysfunction, when senescent cells accumulate within the tissues and SASP activation is persistent. Several studies have provided evidence for the role of senescence and SASP in promoting age‐related diseases or driving organismal ageing. The hype about senescence has been further amplified by the fact that a group of drugs, named senolytics, have been used to successfully ameliorate the burden of age‐related diseases and increase health and life span in mice. Ablation of senescent cells in the brain prevents disease progression and improves cognition in murine models of neurodegenerative conditions. The role of senescence in cancer has been more thoroughly investigated, and it is now accepted that senescence is a double‐edged sword that can paradoxically prevent or promote tumourigenesis in a context‐dependent manner. In addition, senescence induction followed by senolytic treatment is starting to emerge as a novel therapeutic avenue that could improve current anti‐cancer therapies and reduce tumour recurrence. In this review, we discuss recent findings supporting the role of cell senescence in the pathogenesis of neurodegenerative diseases and in brain tumours. A better understanding of senescence is likely to result in the development of novel and efficacious anti‐senescence therapies against these brain pathologies.

## Graphical abstract

This review discusses the role of senescent cells in brain pathologies, in particular age‐associated neurodegenerative diseases such as Alzheimer's disease, Parkinson's disease and multiple sclerosis, as well as brain tumours, specifically craniopharyngioma, low‐grade glioma, glioblastoma multiforme, medulloblastoma and diffuse midline glioma. We present evidence accumulated from in vitro and in vivo studies in both mice and humans. The translational implications of such studies will also be discussed.

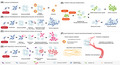

## INTRODUCTION

**FIGURE 1:**
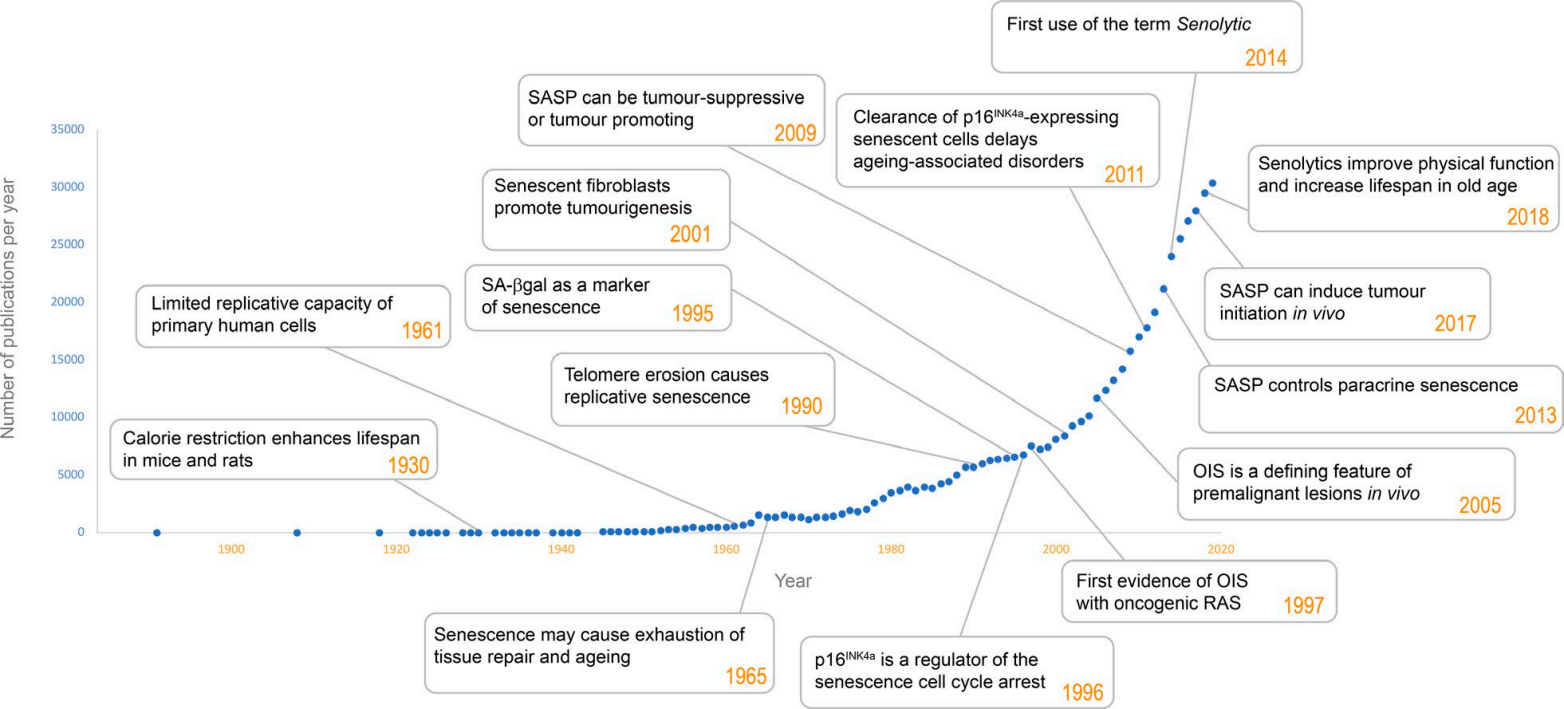
Timeline of research on senescence indicating the number of published papers and milestones

Since the initial discovery and description of senescent cells by Hayflick and Moorhead in 1961 [1], the field of senescence has evolved and expanded immensely. Hayflick originally observed that primary human fibroblast cell cultures had a finite proliferative capacity in vitro. This finite proliferative capacity is now termed replicative senescence and is due to the gradual attrition of telomeres over serial passages [2]. It was initially proposed that replicative senescence was the driver of organismal ageing due to the possible lack of cell replacement and repair [3]. A few decades later, it is now clear that senescent cells are present in many living organisms, from mice to humans and their presence can either be beneficial or detrimental depending on the biological context [4, 5 Figure 1.

Cellular senescence is a survival programme that can be induced by a range of damaging stress signals such as radiation, chemotherapy, replicative stress and oncogenic signalling. Senescent cells are characterised by the stable and irreversible cell‐cycle arrest whilst maintaining metabolic activity and viability [4, 6. This is distinct from cellular quiescence, which is defined as a reversible proliferative arrest, such as adult stem cells, which can be stimulated to re‐enter the cell‐cycle by mitogenic signals [9]. In contrast, senescent cells do not proliferate in respond to these signals. However, they can re‐enter the cell cycle, mostly in the context of developing cancers, whereby accumulation of genetic or epigenetic alterations results in the disruption of the key molecular pathways maintaining cell‐cycle arrest [10, 11, 12, 13.

A hallmark of senescent cells is the activation of a senescence‐associated secretory phenotype (SASP), characterised by the synthesis and secretion of a plethora of biologically active molecules (e.g. inflammatory mediators, growth factors, extracellular matrix proteins) [14, 15, 16. The SASP underpins the paracrine functions of senescent cells. Senescent cells are involved in essential physiological processes such as embryonic development, immune modulation, tissue regeneration, cell plasticity and reprogramming [17, 18, 19. In these contexts, senescent cells are present only transiently to be subsequently eliminated by the immune system [20, 21. In contrast, persistence of senescent cells within tissues results in the deterioration of organ function, which can lead to disease. For instance, age‐related conditions such as osteoarthritis, atherosclerosis, fibrosis of the lungs, kidney and heart, sarcopenia, glaucoma, cataracts and type 2 diabetes are all associated with increased numbers of senescent cells [22, 23, 24. The repertoire of senescence‐associated pathologies has recently been expanded to include neurodegenerative diseases such as Alzheimer's, Parkinson's and multiple sclerosis [25, 26, 27. Moreover, in addition to their role in age‐related diseases, evidence is mounting that accumulation of senescent cells within tissues may be driving organismal ageing itself [8, 28.

**FIGURE 2:**
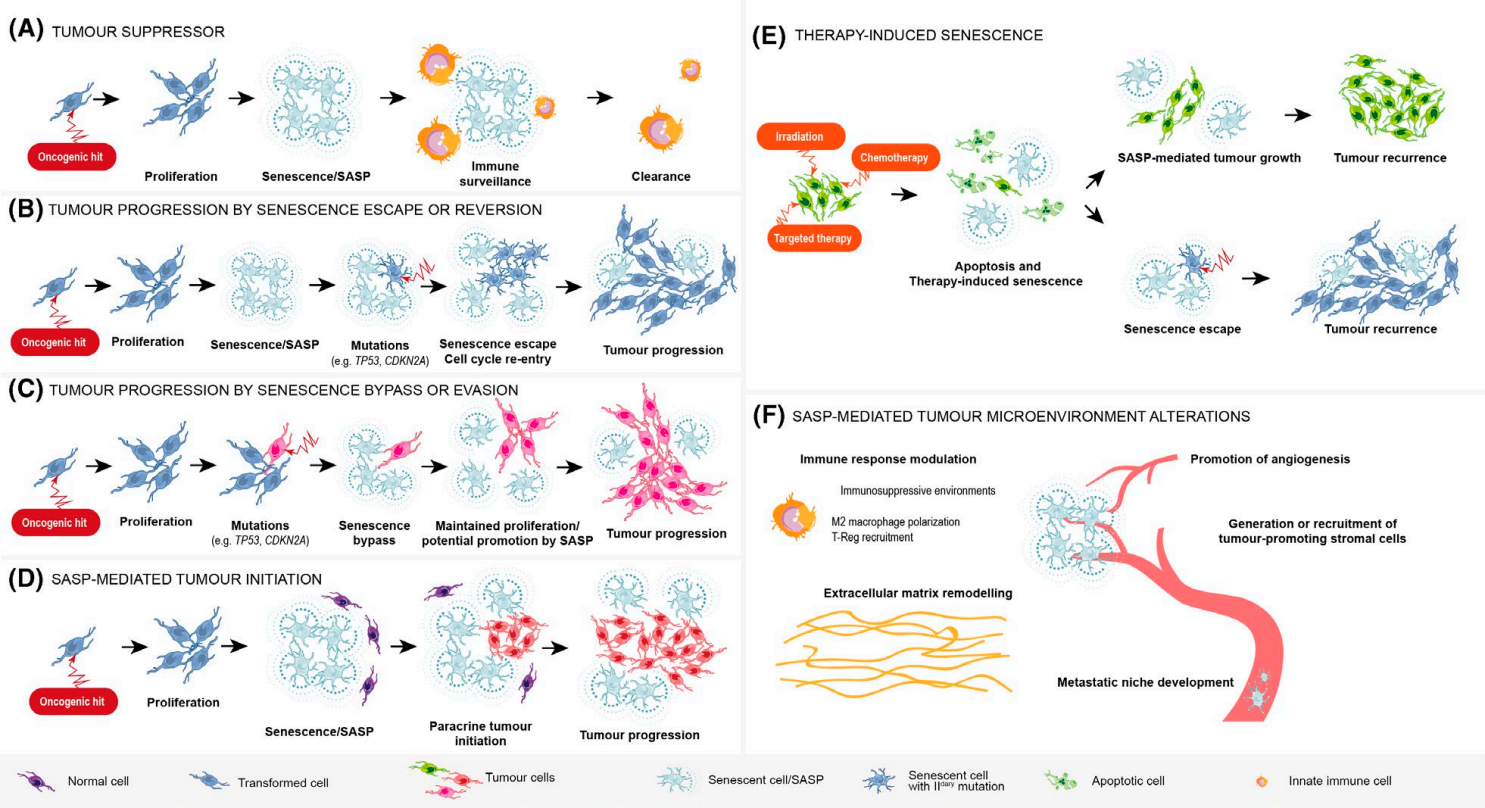
Proposed roles of senescence and SASP in tumourigenesis. (A) Tumour suppressor mechanism. Oncogenic signalling leads to transient proliferation followed by senescence induction (oncogene‐induced senescence). Senescent cells activate SASP and attract immune cells that clear them from the tissues, thus preventing subsequent tumour development. (B) Tumour progression by senescence escape or reversion. Following senescence induction, one cell accumulates further mutations (e.g. *TP53*, encoding p53, or *CDKN2A*, encoding p16^INK4a^) resulting in senescence escape, cell‐cycle re‐entry, proliferation and tumour formation. (C) Tumour progression by senescence bypass or evasion. Upon oncogenic signalling and initial proliferation burst, most of the cells become senescent but one cell continues proliferating due to pre‐existing mutations in key senescence regulators (e.g. p53 or p16) that prevents the establishment of a senescence response. (D) SASP‐mediated tumour initiation. Senescent cells, through the SASP, create a pro‐tumourigenic microenvironment that leads to cell transformation of a non‐tumour cell and tumour formation. (E) Therapy‐induced senescence. Following irradiation, chemotherapy and targeted therapy, most of the cells in the tumour bed are killed (e.g. by apoptosis) or induced into senescence with some cells being unaffected (green cell). Senescent cells will eventually contribute to tumour recurrence either in a paracrine manner through SASP‐mediated tumour growth, or by senescence escape or bypass. (F) SASP‐mediated tumour microenvironment alterations. Senescent cells, through the SASP, can remodel the tumour microenvironment. For instance, by (i) modulating the immune response to create immunosuppressive environment (e.g. M2 macrophage polarisation, T‐reg recruitment), (ii) driving extracellular matrix remodelling and tumour vascularisation and (iii) promoting the development of metastatic niches

Senescence and SASP play a critical role in cancer [36, 37. An early landmark study revealed that the expression of oncogenic RAS, induces premature senescence in primary cell cultures, a process now known as oncogene‐induced senescence (OIS) [38]. This has been further corroborated in different in vivo studies, where oncogenic mutations in different contexts lead to the accumulation of senescent cells [36, 39. Activation of a senescence phenotype constitutes an excellent cell autonomous barrier against development of cancer, by preventing the proliferation of cells harbouring DNA mutations Figure 2A. Cancer progression is understood to require the inactivation of cell‐cycle inhibitors (e.g. p53 and p16^INK4a^) resulting in senescence escape, cell‐cycle re‐entry and tumour cell proliferation [13, 44 Figure 2B. However, this view has been challenged,it has been shown that loss of p53 may not be sufficient for senescence escape, but p53‐defficient cells are able to bypass the establishment of a senescence programme when targeted to express oncogenic mutations [36, 45 Figure 2C. Beyond the cell autonomous role of senescence in cancer prevention and progression through senescence escape or bypass, the paracrine activities of senescent cells can be pro‐tumourigenic Figure 2D. Evidence from in vitro and in vivo studies has demonstrated a critical role of SASP in tumour initiation, progression to malignancy and metastasis [37, 47. In addition to these cell autonomous and non‐autonomous roles of senescent cells in cancer pathogenesis, the senescence response is relevant in the context of cancer therapy. Several standard anti‐cancer treatments such as DNA‐damaging chemotherapy, radiotherapy and even specific targeted therapies against critical pathogenic cancer drivers can trigger therapy‐induced senescence (TIS) in cancer cells or in the microenvironment [51, 52 Figure 2E. New compounds capable of selectively killing senescent cells, termed senolytics, have been identified [5]. These chemical drugs inhibit pathways that are essential for survival of senescent cells but are dispensable in non‐senescent cellular states. Exploiting these new vulnerabilities in cancer is a very exciting area of research, which is likely to lead to novel, efficacious anti‐cancer therapies. Finally, senescent cells can remodel the tumour microenvironment through the SASP creating a permissive setting that allows tumours to progress Figure 2F [37, 53.

In this review we will discuss the role of senescent cells in brain pathologies, in particular age‐associated neurodegenerative diseases such as Alzheimer's disease, Parkinson's disease and multiple sclerosis, as well as brain tumours, specifically craniopharyngioma, low‐grade glioma, glioblastoma multiforme, medulloblastoma and diffuse midline glioma. We will present evidence accumulated from in vitro and in vivo studies in both mice and humans. The translational implications of such studies will also be discussed. For further reading, we recommend other reviews in the field [4, 5, 37, 54.

## CELLULAR SENESCENCE AND SASP

The permanent cell‐cycle arrest of senescent cells is mediated principally by the p16^INK4a^/Rb and p21^Cip1^/p53 pathways in response to stress stimuli [56, 57, 58, 59. Expression of p53 due to cellular stress signals activates a multitude of responses, including cell‐cycle arrest, which is mediated by the p53 target p21^Cip1^. p16^INK4a^ mediates cell‐cycle arrest by inhibiting CDK4/6 leading to hypo‐phosphorylation of RB and inhibition of cell‐cycle progression into S phase. p16^INK4^ has been termed the master regulator of cell‐cycle arrest in senescent cells [57, 58. Activation of the senescence programme leads to further molecular changes: (i) chromatin remodelling (e.g. presence of senescence‐associated chromatin foci), (ii) activation of a DNA damage response (e.g. expression of γH2AX), (iii) enlargement of the lysosomal compartment (e.g. increased expression of GLB1 and lipofuscin accumulation); (iv) macromolecular damage (e.g. telomere attrition); (v) deregulated metabolism (e.g. a shift from oxidative phosphorylation to glycolysis); (vi) anti‐apoptotic response (e.g. increased expression of BCL‐2 family proteins and inhibition of caspase 3) [4, 6. Resistance to apoptotic death is primarily mediated through the stress‐induced p53 pathway, which upregulates the expression of anti‐apoptotic BCL‐2 proteins [60, 61. Additionally, the p53‐transcriptional target p21^Cip1^ has been shown to be able to directly inhibit caspase 3, hence contributing to apoptotic resistance [62].

A main feature of senescent cells is the activation of the SASP [63, 64, 65. SASP factors include cytokines and chemokines (e.g. IL1A, IL1B, IL6, L8, CCL2, CCL5, CCL20 TGFβ and TNFα), growth factors (e.g. EGF, bFGF, HGF, VEGF), proteases (e.g. MMP‐1, −3, −10, −12, −13, −14), extra cellular matrix components (e.g. fibronectin, collagens, laminin) and non‐protein components such as lipids and prostaglandins [36, 63, 66. The SASP is dependent on several signalling pathways such as NFκB, p38MAPK, mTOR, NOTCH, C/EBPβ and activation of the cGAS‐STING pathway [68, 69, 70, 71, 72, 73, 74. There is no unique SASP, on the contrary the composition and intensity of the SASP depends on the cell type, the senescence‐inducing stimulus and the timing after SASP activation [75]. SASP factors reinforce the senescent phenotype by autocrine or paracrine signalling, but can also affect the microenvironment via paracrine signalling [53, 64. Senescent cells can also affect their neighbouring cells via juxtacrine NOTCH signalling or by the transfer of cellular cargo by either cytoplasmic bridges or exosome production [14]. The consequences of SASP activation can be either beneficial or detrimental depending on the biological context. For example, senescent cells in skin secrete the SASP factor PDGF‐AA upon wounding, which is important for optimal healing [76]. Furthermore, senescent cells and the expression of SASP can contribute to the dedifferentiation and reprogramming on non‐senescent adjacent cells [19]. Dysregulated and chronic SASP is detrimental and can cause age‐related diseases, organismal ageing and cancer[37].

The identification of senescence in vivo is difficult, and no single marker can unambiguously be used to define senescent cells. Initially the expression of β‐D galactosidase and its detection in a colorimetric enzymatic assay at acidic pH (SA‐β‐Gal) was used to define senescent cells. However, this staining can be unreliable in vivo. Therefore, a consensus has been published, where a combination of markers is recommended to assess cellular senescence [5] Table 1.

**TABLE 1:** Detection of senescence: senescence hallmarks, markers, biological consequences and limitations

Senescence hallmarks	Markers	Limitations	Biological consequences
Cell‐cycle withdrawal	Ki67 negativity, phopho‐histone H3 negativity, EdU exclusion	Also in quiescence	Irreversible cell‐cycle arrest
	p21^Cip1^ positivity, *CDKN1A* upregulation	Also in quiescence	CDK2 inhibitor p21^Cip1^ accumulation
	p16^INK4a^ positivity, *CDKN2A* upregulation	Express by non‐senescent cells (macrophages), not express by all senescent cells	CDK4/6 inhibitor p16^INK4A^ accumulation
	p15^INK4b^ positivity, *CDK2B* upregulation		Other cyclin inhibitors accumulation
	Cyclin‐A2 *(CCNA2)* and Cyclin‐E2 (*CCNE2)* downregulation		Decreased expression of cyclins
	Persistent activation of RB family proteins (e.g phosphorylation of RB1, p107, p130)	Also in quiescence	Stability of the senescent state
	Heterochromatinization of E2F target genes		
Macromolecular damage	Telomere shortening		DNA Damage
	Persistent nuclear DNA damage foci		
	Expression of γ‐H2AX and PARP‐1		
	Phosphorylation of DNA‐PKcs and ATM		
	Ubiquitin proteasome system active		Protein Damage
	Autophagy		
	Fatty acids and free cholesterol increase/phospholipids and cholesteryl decrease	High variability of the senescence‐associated lipid profile	Lipid Damage
Secretory phenotype (SASP)	NF‐κB, C/EBPβ, GATA4, mTOR and p38MAPK signaling pathways (phosphorylation of IkBa, p38,…)	High Variability: duration, cell type, inducer stimuli, and cell‐to‐cell variability	Activation of transcription factors
	IL6; IL7; IL1; IL1B; IL13; IL15 ; TGFβ; GM‐CSE; G‐CSE; IFN‐γ; BLC; MIF		Pro‐inflammatory cytokines release
	IL8; GRO‐a, ‐b, ‐g; MCP‐2; MCP‐4; MIP‐1a; MIP‐3a; HCC‐4; eotaxin; eotaxin‐3; TECK; ENA‐78; I‐309; I‐TAC		Chemokines production
	Amphiregulin; epiregulin; heregulin; EGF; bFGF; HGF; KGF (FGF7); VEGF; angiogenin; SCF; CXCL12; PIGF; NGF; IGFBP2, IGFBP3, IGFBP4, IGFBP6, IGFBP7		Growth modulators, angiogenic factors
	MMP‐1, ‐3, ‐10, ‐12, ‐13, ‐14; TIMP‐1; TIMP‐2; PAI‐1, ‐2; tPA; uPA; cathepsin B		Proteases, matrix metalloproteinases
	ICAM‐1, ‐3; OPG; sTNFRI; sTNFRII; TRAIL‐R3; Fas; uPAR; SGP130; EGF‐R ; Fibronectin; collagens; laminin		Secretion of other factors
Deregulated metabolism	Increase number, decreased membrane potential, increased proton leak		Less functional mitochondria
	PML nuclear bodies (isoform IV)	Also during apoptosis	Reactive oxygen species (ROS) production
	Senescence‐associated beta‐galactosidasde (SA‐β‐gal) activity	Not required for the senescent phenotype	Lysosomes increase in number and size
	Galactosidase, beta 1 *(GLB1)* upregulation		
	LAMP1, LAMP2, Lysozime C upregulation		
Senescence‐associated epigenetic	H4K16ac, H3.3, H4K20me3 and H3K9me3		Global increase in chromatin accessibility
	Senescence‐associated heterochromatin foci (SAHFs)		
	Global loss of linker histone H1		
	Lamin B1 (*LMNB1*) loss and reduced nuclear integrity		
	Upregulation of specific miRNAs (e.g. miR‐504, miR‐605)		Change in miRNAs expression
Resistance to apoptosis	Increased expression of BCL‐2 family members		Anti‐apoptotic protein upregulation
	TRAIL‐Decoy Receptor DcR2 over expression	Markers not present in mice	Hiding from Immune system
	NKG2D ligands over expression		

## ANTI‐SENESCENCE AND ANTI‐SASP STRATEGIES

After the discovery of the detrimental role that senescent cells play in ageing and in numerous pathologies, it soon became relevant to develop specific targeted strategies. The first proof‐of‐concept that the ablation of senescent cells was beneficial and reduced ageing‐associated disorders was published in 2011 [28, 77. This was followed by studies showing that the selective killing of senescent cells using chemical compounds improves organ function in ageing mice [33, 78. A non‐exhaustive list of current and promising strategies is presented in Table 2. There are four main approaches of anti‐senescence and anti‐SASP strategies currently being investigated.

**TABLE 2:** Anti‐senescence and anti‐SASP strategies: target proteins, agents and clinical development status

Candidate senotherapy	Target class/target	Agent	Class	Development status		
				*In vitro* inducer	Preclinical models	Clinical trials
Senolytics	Pan‐kinase	Quercetin	Small inhibitor	Replicative Senescence		Phase II Chronic Kidney Disease NCT02848131
	Pan‐receptor tyrosine kinase	Dasatinib	Small inhibitor	Replicative Senescence		Phase II Skeletal Health in aging NCT04313634
	Pro‐survival proteins BCL‐2, BCL‐XL, BCL‐W	ABT‐737	Small inhibitor	Replicative Senescence Oncogene Induced Senescence Irradiation, Cytostatic drugs		
		A1155463	Small inhibitor			
		A‐1331852	Small inhibitor			
		ABT‐263 (Navitoclax)	Small inhibitor			
	MDM2/p53 protein interaction	UBX0101	Small inhibitor	Replicative Senescence	Osteoarthritis mouse model	Phase II Knee Osteoarthritis NCT04129944
		P5091, P22077	USP7 inhibitor	Replicative Senescence Irradiation, Cytostatic drugs	Doxorubicin‐treated mice	
	FOXO4	FOXO4 peptide	Peptide blocking interaction with p53	Replicative Senescence Irradiation, Cytostatic drugs	XpdTTD/TTD mice, p16‐3MR aged mice	
	HSP90	Alvespimycine (17‐DMAG)	Small inhibitor, antibiotic	Replicative Senescence Irradiation, Oxidative stress	Ercc1 ‐/∆ progeroid mice	
		Ganetespib	Small inhibitor			
	Unknown	Fisetin	Flavonoid polyphenol	Replicative Senescence	Ercc1 ‐/∆ progeroid and old INK‐ATTAC mice	Phase II Frail Elderly Syndrome NCT03675724, NCT03430037
		Luteolin	Flavonoid polyphenol	Replicative Senescence		Phase I/II Osteoarthritis NCT04210986
						Phase II Chronic Kidney Diseases, Diabetes NCT03325322
						Phase II Skeletal Health in aging NCT04313634
	Na+/K+ ATPase	Ouabain	Cardiac Glycosides	Replicative Senescence Irradiation, Cytostatic drugs.	Lung adenocarcinoma, melanoma xenograft	
					Liver cancer, ACP mouse models	
		Digoxin			Irradiated mice	
					Ageing mice	
	Mitochondria	MitoTam	Mitochondria‐targeted tamoxifen	Replicative Senescence, Cytostatic drugs	Ageing mice	
Vectorisation	Encapsulation of drugs with galacto‐oligosaccharides	Mesoporous Silica Nanoparticles		Oncogene Induced Senescence, Cytostatic drugs	Melanoma xenograft	
		*Multiple cargo: doxorubicin, navitoclax,*			Triple negative breast cancer xenograft	
					Fibrotic lungs mouse model	
	Galactose‐modified prodrug	Duocarmycin derivatives		Replicative Senescence, Oncogene Induced Senescence Irradiation Cytostatic drugs	ACP mouse model	
					Irradiated mice	
		Navitoclax		Oncogene Induced Senescence Irradiation, Cytostatic drugs	NSCLC mouse models	
SASP prouction modulators	IKK, NF‐κB and DICER	Metformin	Small inhibitor	Replicative Senescence, Oncogene Induced Senescence		Approved for Diabetes
	mTOR	Rapamycin	Small inhibitor	Replicative Senescence, Irradiation, Cytostatic drugs	Ageing mice	Approved immunosuppressor
	JAK‐STAT	Ruxolitinib	Small inhibitor	Replicative Senescence	Ageing mice	Approved immunosuppressor
	Glucocorticoids Receptor	Corticosterone, Cortisol	Glucocorticoids	Irradiation		Approved immunosuppressor
	p38MAPK		Small inhibitor			
SASP blocking antibodies	IL1A, IL1B, IL1Receptor	Rilonacept, Canakinumab, Anakinra				Clinicaly approved immunosuppressors
	TNF	Etanercept, Infliximab				
	IL6, IL6R	Siltuximab, Tocilizumab				
Immunotherapies	Antibody‐dependent cell‐mediated cytotoxicity with NK cells	DPP4 antibody	Antibody	Replicative Senescence		
	NKG2D CAR‐T cells					
Calories restriction mimetics		2‐deoxy‐D‐glucose	Glucose analogue	Replicative Senescence Oncogene Induced Senescence		
		Resveratrol	Polyphenol	Replicative Senescence Oncogene Induced Senescence	Ageing mice	

### Prevention of senescent cell accumulation

The chronic reduction of total calorie intake has been reported to counteract several age‐associated alterations, through molecular and physiological effects, including prevention of senescent cells accumulation [80, 81, 82. As a consequence, caloric restriction mimetics are studied in the context of ageing, particularly among them the modulation of glucose metabolism by 2‐deoxy‐D‐glucose, which has been shown to reduce degeneration of dopaminergic neurons in a Parkinson's disease mouse model [83]. Resveratrol and other polyphenols are also able to suppress the formation of reactive oxygen species (ROS) and to limit cellular senescence in neurons [84, 85. Cells treated with caloric restriction mimetics express molecular pathways similar to cells affected by long‐term calorie restriction or short‐term fasting, including the autophagy pathway. The crosstalk between autophagy and SASP production is an important element to investigate to better understand the regulation of cell senescence by these drugs.

### Ablation of senescent cells: senolytics

Among the senescence hallmarks, the anti‐apoptotic programme is not only required for senescent cell survival, but also the easiest to target. Thus, the first senolytic drugs that have been reported are inhibitors of the anti‐apoptotic B cell lymphoma 2 (BCL‐2) protein family [33, 78. Two of these promising drugs, ABT‐263 and ABT‐737, have been shown to be capable of selective elimination of senescent cells and causing therapeutic benefits in several physiological and disease contexts (e.g. regeneration [86], cancer [87], type 1 diabetes [88], and atherosclerosis [89]. Other anti‐apoptotic pathways have been investigated, in particular the inhibition of the MDM2/p53 interaction (e.g. UBX0101 [90] and USP7 inhibitor [91]. In mouse models, UBX0101 is able to attenuate the development of osteoarthritis by selective clearance of senescent cells, however a phase II trial did not replicate these results (NCT04129944) [92]. Recently, high throughput drug screenings have uncovered the senolytic activity of cardiac glycosides, through a process mediated by induction of the pro‐apoptotic BCL2‐family protein NOXA [93, 94. Another class of senolytics take advantage of the high lysosomal β‐galactosidase activity of senescent cells to deliver more specifically cytotoxic drugs to senescent cells and reduce the toxic side effects [95, 96.

### Making senescent cells harmless: SASP‐modulating drugs

An additional approach to interfere with the detrimental effects of senescent cells is the modulation of their secretome, either by disrupting the overall production, maturation or secretion of SASP factors, or by the selective blockade of specific components. The NF‐κB inhibitor Metformin, the mTOR inhibitor Rapamycin and the JAK/STAT inhibitor Ruxolitinib have been shown to suppress SASP activation by inhibition of critical pro‐SASP cascade signalling [71, 97. The blockade of specific SASP factors (e.g. IL1B and IL6) or their receptors has the potential to reduce off‐target effects, and anti‐inflammatory drugs inhibiting these factors have already been clinically approved (e.g. Tocilizumab against the IL6 receptor, Anakinra against IL1B). However, the redundancy and pleiotropic functions of SASP factors may make it difficult to target SASP therapeutically and no trials have yet been successfully conducted using this strategy.

### Enhancing organismal anti‐senescence systems: immune clearance

Senescence activates the innate and adaptive immune responses, which result in elimination of senescent cells in physiological contexts (i.e. immune clearance). However, decline of immune system surveillance associated with ageing or immunosuppressive microenvironments results in immune evasion of senescent cells leading to tissue accumulation and subsequent deterioration of organ function [20, 99. Boosting immune surveillance through the use of biotherapeutics, such as engineered immune cells (e.g. chimeric antigen receptor T (CarT) cells or natural killer cells) has been successfully used for the treatment of human cancer [102]. Similar approaches could be effective against membrane‐bound proteins that are present in senescent cells (e.g. uPAR [103] and DPP4) [104], although toxicity may hamper the potential therapeutic use of this strategy.

More research is needed to identify safe and efficacious anti‐senescence therapies able to counteract the detrimental effects of ageing and cancer. As previously mentioned, senescent cells are highly heterogeneous and the activation of specific transcriptomic programmes is dependent on cell type, stress inducers and duration of senescent induction [75, 105. Therefore, the identification of the best anti‐senescence approach may need to be tailored to the specific cellular context, whether ageing, specific degenerative disease or cancer.

## SENESCENCE IN NEURODEGENERATIVE DISEASES

Age is the most common risk factor for neurodegenerative diseases [106]. The incidence of conditions such as Alzheimer's and Parkinson's disease, which are characterised by cognitive decline and loss of neurons and synaptic connections, increases with age [107]. Age is also a risk factor for inflammatory diseases such as multiple sclerosis, which show loss of axons, dendrites, and neurons [108]. Senescent cells have been identified in different cell types of the nervous system, including neural stem cells, neurons, astrocytes, oligodendrocytes and microglia [109, 110, 111, 112, 113, 114, 115. Although neurons are characterised by permanent exit of the cell cycle, they have been shown to accumulate DNA damage and acquire additional features that typify senescence, including SASP activation [113]. These senescent cell types have been implicated in the pathogenesis of Alzheimer's disease, Parkinson's disease, multiple sclerosis, frontotemporal dementia and ischaemia/stroke. Cellular senescence may contribute to the initiation and/or progression of neurodegenerative diseases by promoting chronic inflammation, causing loss of regenerative properties and enhancing age‐related decline in the blood‐brain barrier and micro‐vasculature [116].

### Alzheimer's disease

Alzheimer's disease (AD) is the most common neurogenerative disease with an incidence of 11.08 per 1000, doubling every 5 years after 65 years of age. AD affects more than 35 million people worldwide. The main pathological features that define AD are an accumulation of Aβ peptide amyloid plaques and neurofibrillary tangles (NFTs) of hyperphosphorylated tau proteins in the hippocampus and cerebral cortex [117]. Additional features include loss of neurons and synapses in the dentate gyrus of the hippocampus and cerebral cortex resulting in progressive cognitive decline and memory loss [118]. Senescent cells have been identified in both AD human samples and mouse models opening the possibility that these cells contribute to the pathogenesis. Supporting this statement, expression of p16^INK4a^ and p53 are elevated in post‐mortem human AD samples compared with age‐matched control brains [119, 120.

Progressive loss of neurons and neural stem cells in the dentate gyrus of an ageing hippocampus may contribute to the aetiology of AD. Neural stem cell senescence could explain the loss of neural progenitor proliferation that is observed in both, mouse models of AD and premature ageing, as well as in human brains from old individuals [121, 122, 123, 124. Recent experiments in vitro have demonstrated the presence of senescent neural stem cells in AD. The formation of Aβ oligomers has been shown to induce senescence in hippocampal neural stem cells of the APP/PS1 AD mouse model [125]. In this study, Aβ fibrils can accelerate neural stem cell senescence via activation of the MAPK pathway, ultimately leading to loss of neurogenesis.

Higher levels of pro‐inflammatory SASP factors has been reported in aged human and mouse brains compared with younger controls [126]. Inflammation is a key feature which contributes to the initiation, severity and progression of most neurodegenerative diseases including AD [127]. Expression of SASP factors, e.g. IL6, IL1B, TGFβ, TNFα and MMP‐1, −3 and −10 and activation of the p38MAPK pathway are upregulated in human AD samples and murine models [72, 128.

Microglia, the resident macrophages of the central nervous system (CNS) whose functions are tightly regulated by their microenvironment, can secrete SASP factors [133]. Ageing or neurodegenerative accumulation of misfolded protein induces microglia proliferation and promotes an activated state. This state is known as microglia priming and initiates the reactive defence program characterised by phagocytosis and increased release of cytokines, tumour necrosis factor (TNF) and nitric oxide [134]. Primed microglia are also prone to be stimulated by secondary sources of inflammation, triggering an exaggerated and chronic inflammatory response in the CNS [135]. Both aged and AD brain samples show microglia priming and an increase of their pro‐inflammatory response [134].

Ex vivo and in vitro studies have revealed that aged microglia secrete higher levels of SASP factors such as IL6 and TNFα compared with young microglia. Aged microglia lose their ability to phagocytose Aβ fibrils and undergo replicative senescence due to telomere shortening [110, 136. Evidence linking age‐related senescence and AD pathogenesis has been provided by a study in which aged rat microglia were isolated and treated with Aβ oligomers in vitro. Upon treatment, these activated microglia become senescent, shown by SA‐βgal staining and production of IL1B, TNFα and MMP2 [137]. This suggests that age‐related senescence in AD microglia may play a role in disease progression.

Primed microglia and neuroinflammation are considered to play key roles in the initiation and progression of AD. An increase in numbers of primed microglia correlates with AD disease progression in humans [138], however, the mechanisms by which these cells could detrimentally affect AD pathogenesis are not yet fully elucidated. Primed microglia release IL1B and IL18 [139], and in a study on human AD, it has been shown that IL1B induces the secretion of TNFα, promoting the formation of amyloid plaques [140].

Another heterogenous cell population implicated in AD are astrocytes [141]. These cells have diverse homeostatic roles in the CNS including neurotransmitter uptake/recycling, synaptic activity, maintenance of the blood brain barrier and inflammation [142]. Single cell sequencing from wild type and AD mouse models has identified disease‐specific astrocytes that are apparent before the onset of neurological phenotypes and are increased with disease progression [138]. These astrocytes express an inflammatory and neurotoxic gene profile that is analogous to that observed in aged wild‐type astrocytes (i.e. upregulation of genes involved in development and differentiation, metabolic pathways of lipid and cholesterol, response to toxic compounds and inflammatory signalling, including NfκB signalling and ROS). Furthermore, these upregulated genes have been identified in aged human brain samples from AD post‐mortem samples, confirming previous studies in which overexpression of IL6 in murine astrocytes results in the appearance of AD‐like neurological symptoms [143] and in the formation of amyloid plaques that are similar to those observed in human AD patients [144, 145. These studies provide evidence that neuroinflammation, caused by the secretion of chemokines and cytokines commonly found in the SASP, contributes to the initiation and progression of AD.

#### Senolytic therapy in AD

A recent report has identified senescent oligodendrocyte precursors (OPCs) (a subset of glial cells in the brain) in human AD and in the APP/PS1 AD murine model [26]. These senescent cells are associated with Aβ plaques in both species and the study suggests that Aβ fibrils trigger OPC senescence. The senescent cells in vivo are positive for SA‐β gal staining, co‐express OPC markers together with p16^INK4a^ and p21^Cip1^, and show increase levels of *CDKN2A* (encoding p16^INK4a^) mRNA. Acute oral administration of Dasatinib and Quercetin (a senolytic drug combination) selectively kills senescent OPCs in the Aβ plaques, consequently reducing IL6 levels. The selective killing of these senescent cells not only reduces neuroinflammation but decreases Aβ loads and ameliorates the AD cognitive defects [26]. A phase II clinical trial has been initiated in patients with AD using this senolytic combination [146].

In a study using micro‐dissected post‐mortem human AD, a senescent transcriptomic profile has been identified in neurons containing neurofibrillary tangles (NFTs) of aggregated tau protein [147]. NFT‐accumulating neurons in different AD murine models display a senescent phenotype, evidenced by expression of *CDKN2A* mRNA. Treatment with Dasatinib and Quercetin can kill these senescent cells resulting in reduction of both NFT density and neuronal loss.

Collectively, these findings indicate a strong association between the presence of cellular senescence in the brain and neurodegeneration, which is supported by mechanistic studies in murine models. Potential therapeutic avenues that selectively kill senescent cells could revolutionise AD treatments.

### Parkinson's disease

Parkinson's disease is the second most common neurodegenerative disease after AD, affecting about 7–10 million people worldwide over the age of 65. It is characterised by the progressive loss of dopaminergic neurons in the *substantia nigra pars compacta* of the midbrain, leading to progressive motor degeneration. A key pathological feature is the presence of Lewy bodies, composed of aggregates of α‐synuclein, a protein involved in DNA damage repair [148]. PD symptoms manifest once 80% of the dopaminergic neurons are lost [149]. Currently there are no chemical treatments that can prevent disease progression.

Evidence of senescence in PD has been shown in various studies. Higher expression levels of p16^INK4a^, p21^Cip1^ and inflammatory markers (such as IL6) have been identified in PD patients compared with healthy controls. Increased expression of these factors is associated with faster cognitive decline in the patients [27]. A recent study has revealed that a DNA binding protein, STAB1, which is associated with PD, prevents cellular senescence in dopaminergic neurons in vivo [150]. Loss of STAB1 in human stem cell‐derived dopaminergic neurons causes activation of a senescence programme in vitro. Inhibition of STAB1 in human PD brain slice cultures or loss of STAB1 in mice also activates a senescence programme. STAB1 directly represses *CDKN1A* (encoding p21^Cip1^) in DA neurons and activates a senescence and SASP programme. Expression levels of p21^Cip1^ in DA neurons in the *substantia nigra* are elevated compared with age‐matched controls. Increased p21^Cip1^ protein levels, potentially suggesting senescence, have also been observed in neural stem cells in the Parkin‐deficient (*Prkn* ^−/−^) PD mouse model. Parkin is an E3 ubiquitin ligase needed for targeted protein degradation, which is essential for neurogenesis. Protein ubiquitination plays a key role in neural stem cell renewal and differentiation and impaired neurogenesis is found in PD [151].

A common feature of PD is the presence of activated microglia and astrocytes, which could contribute to chronic neuroinflammation. Elevated levels of inflammatory factors have been detected in the cerebrospinal fluid (e.g. IL1B) [152] as well as in DA neurons (e.g. IL1B and IL6) [153] in PD patients. Post‐mortem examination of PD samples has revealed the expression of senescence and SASP markers such as p16^INK4a^, IL6, IL1A, IL8 and MMP3. Dysfunctional lysosomes and increased SA‐βgal staining has also been identified in human PD samples [154, 155.

Senescent astrocytes have been observed in both human PD samples and a PD murine model [115]. Sporadic PD in humans has been associated with exposure to the herbicide paraquat (PQ), and PQ administration to mice is sufficient to induce PD‐like phenotypes. Human astrocytes cultured in vitro with PQ show positive SA‐βgal staining and reduced proliferation. Conditioned medium from these senescent astrocytes reduces human DA neurone viability and decreases neural stem cell proliferation [115]. The genetic ablation of p16^INK4a^‐expressing senescent cells in the context of a PQ‐induced PD mouse model is sufficient to abrogate PD‐associated motor deficits and neuropathology. Together, there is evidence supporting the presence of disease‐relevant senescent cells with an activated SASP in human and murine PD, providing a rationale for the therapeutic targeting of these cells.

### Multiple sclerosis

Multiple sclerosis (MS) is an autoimmune disease causing severe physical incapacitation and neurological damage, affecting around 2.5 million people worldwide [156]. The debilitating causes of MS are due to CNS demyelination and neurodegeneration with limited remyelination. Infiltrating macrophages and lymphocytes cause multifocal inflammation and oligodendrocyte cell death, which lead to demyelination, neuronal and axonal loss, and generation of CNS plaques that contain inflammatory cells and demyelinated axons. The aetiology of MS remains unknown, but certain genetic and environmental factors might influence the likelihood of developing MS [108]. Several immunosuppressive and immunomodulatory treatments are available,however, disease progression is still common.

The presence of senescent cells with activated SASP has been observed in mouse models and human MS samples. Using a gliotoxin‐induced demyelination MS murine model, it has been reported that aged mice show slower rates of remyelination than younger mice, suggesting that age‐related senescence could play a role in the onset and progression of this disease [157]. In another mouse model, in which demyelination is induced by feeding the mice cuprizone, increased numbers of SA‐βgal‐positive glial cells have been identified in demyelinating fibres of the *corpus callosum* [158]. In comparison with age‐matched control tissue, demyelinated human MS lesions show increased numbers of SOX2+ cells (a marker of neural progenitors) co‐expressing p16^INK4a^, suggesting the presence of senescent progenitor cells in progressive MS [159]. This finding has been corroborated in in vitro differentiation experiments of iPSC lines derived from either MS patients or age‐matched controls. For instance, expression of senescent markers (e.g. SA‐βgal staining, p16^INK4a^ and p53) is elevated in iPSC‐derived neuronal progenitor cells (NPCs) from MS patients relative to healthy controls. Interestingly, the inhibition of the mTOR pathway with rapamycin reverses the senescent phenotype and results in reduced SA‐βgal staining and p16^INK4a^ expression levels in iPSC‐derived NPC from MS patients. Further evidence that SASP activities may be involved in MS comes from in vitro studies assessing the capacity of senescent NPCs to promote differentiation of oligodendrocyte progenitor cells (OPCs) into myelinating oligodendrocytes (MOs). Conditioned medium from healthy control‐derived NPCs can induce differentiation of OPCs into MOs in vitro, whilst MS‐patient‐derived NPCs conditioned medium fails to induce differentiation. However, rapamycin treatment of NPCs from MS patient‐derived NPCs yields conditioned medium with comparable OPC to MO differentiation potential to that of healthy control‐derived NPCs. These findings suggest that paracrine signals from MS‐patient‐derived NPCs inhibit OPC to MO differentiation, which can be reversed by inhibition of mTOR, a critical pathway maintaining SASP activation. Proteomic analysis of conditioned medium from MS‐patient‐derived NPCs has shown the presence of secreted proteins previously associated with cellular senescence, such as heat shock proteins 90 and 60, DJ‐1, and HMGB1 and a similar molecular profile to aged NPCs derived from healthy controls.

Currently there is no cure for MS, treatments so far rely on immunomodulation. However, the identification of senescent cells and SASP factors in MS patients and murine models supports the possibility that senescence may play a role in the pathogenesis of MS, hence providing a rationale for the specific targeting of these cells using senolytics.

## SENESCENCE IN BRAIN TUMOURS

Senescent cells play an important role in tumourigenesis and can act as a double‐edged sword. On one hand, senescence limits proliferation of cells bearing DNA damage in a cell autonomous manner, thus preventing tumour progression, which can occur only by senescence bypass or escape. Paradoxically, senescent cells through the SASP can generate a pro‐tumourigenic microenvironment that fuels tumour initiation and progression, including senescence escape or bypass. Additionally, standard anti‐cancer treatments (e.g. chemo‐, radio‐ and targeted therapies) can effectively trigger therapy‐induced senescence (TIS) to create new vulnerabilities in tumour cells that could be exploited using senolytics and/or SASP modulators. Here, we will discuss the role of senescent cells in brain tumours.

### Craniopharyngiomas

Craniopharyngiomas (CPs) are benign epithelial tumours (WHO grade 1) of the sellar region (an anatomical region between the hypothalamus and the cranial base where the pituitary gland is located). There are two types: (i) adamantinomatous (ACP), which carry mutations in *CTNNB1* (encoding β‐catenin) resulting in the activation of the WNT/β‐catenin pathway; (ii) papillary (PCP), associated with mutations in *BRAF*‐V600E leading to the activation of the MAPK/ERK pathway. Although these tumours are associated with high survival (over 90% 5 years survival), they cause significant morbidity and poor quality of life for the patients, in particular ACPs due to their tendency to invade the hypothalamus and optic chiasm [160, 161.

**FIGURE 3:**
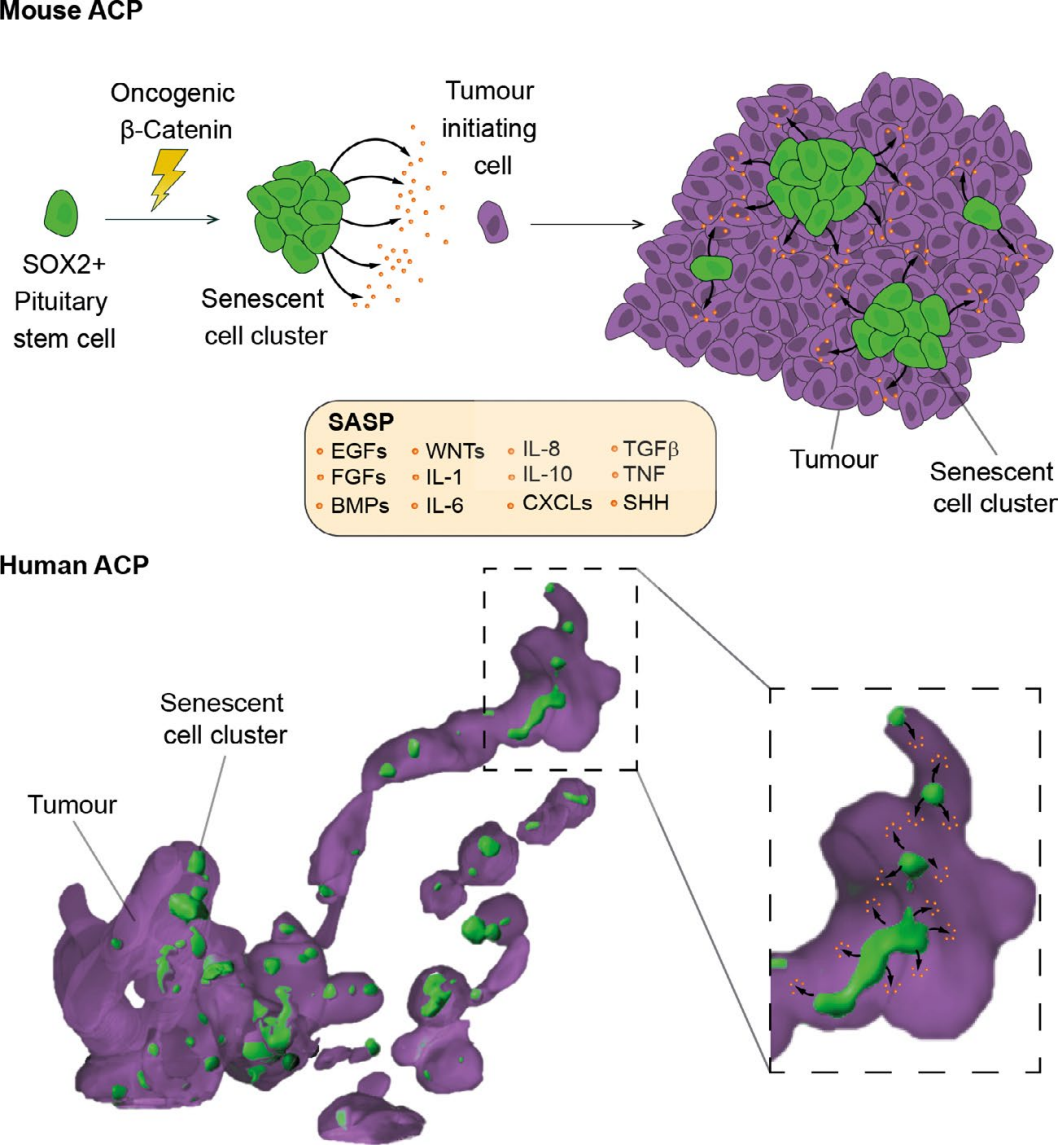
Schematic showing a working model for the role of the β‐catenin‐accumulating cell clusters in mouse and human ACP. (A) Expression of oncogenic β‐catenin in SOX2+ pituitary stem cells (both embryonic and postnatal) results in the formation of β‐catenin‐accumulating cell clusters, which contain senescent cells (oncogene‐induced senescence). Senescent cluster cells activate a senescence‐associated secretory phenotype (SASP), which leads to the synthesis and secretion of a plethora of active peptides, some of which are included in the box. The persistent activity of the SASP factors on surrounding cells eventually causes cell transformation of a cell not of the SOX2 cell lineage (purple cell) and subsequent tumour development in a paracrine manner. (B). The human tumour depicted in the schematic derives from a three‐dimensional reconstruction of a micro‐CT‐imaged human ACP sample, in which the glial reactive tissue has not been rendered. Purple indicates the stellate reticulum and cells of the palisading epithelium, and green represents the β‐catenin‐accumulating cell clusters. Note the presence of finger‐like protrusions of tumour cells, which project away from a tumour epithelium mass, containing a string of clusters inside. These human clusters are molecularly analogous to the mouse clusters and share a signature of senescence and SASP. The model proposes that the SASP activities underlie tumour growth and invasive behaviour by promoting epithelial remodelling and proliferation. Reproduced with permission from S. Karger AG, Basel (Martinez‐Barbera J. P. and Andoniadou C. L., Biological Behaviour of Craniopharyngiomas. Neuroendocrinology 2020. https://doi.org/10.1159/000506904)

Senescence has not been characterised in PCP; however, a bulk of research has demonstrated the existence of senescent cells in human ACP. Despite the presence of *CTNNB1*‐activating mutations in all the tumour cells, [162] accumulation of nucleocytoplasmic β‐catenin and activation of the WNT pathway occurs in sporadic cells, most of which form groups of cells referred to as clusters. These cell clusters are not present in PCP or any other pituitary tumours. Immunohistochemistry has demonstrated that these cluster cells show the hallmarks of senescence: they are Ki‐67‐ve, express cell‐cycle inhibitors (e.g. p21^Cip1^), show DNA damage (e.g. γ‐H2aX staining), activate the DNA damage response (e.g. phospho‐DNA‐PKc staining), exhibit enlargement of the lysosomal compartment (e.g. GLB1 expression) and turn on the NFκB pathway (phospho‐IκB staining) [48, 163. Laser capture microdissection followed by transcriptomic analysis has confirmed that human cluster cells are senescent and activate a SASP resulting in the expression of numerous inflammatory mediators and growth factors [48, 162, 164. The location of the clusters within the finger‐like protrusion invading the brain strongly suggests that the paracrine activities of the cluster cells may play a role in tumour epithelium remodelling, proliferation and invasion [165] Figure 3.

These findings in human ACP have been confirmed in ACP mouse models. Genetically modified mouse models of ACP have been generated by expression of a functionally equivalent mutant form of β‐catenin in SOX2+ve stem cells of the pituitary gland during embryonic development and adulthood [166, 167. Like human ACP, the mouse tumours contain cell clusters expressing senescence markers. Interestingly, genetic tracing in the ACP mouse models has revealed that the clusters derive from SOX2+ve stem cells expressing oncogenic β‐catenin. In contrast, the tumours are derived from a different cell lineage and do not express oncogenic β‐catenin [167]. This initial study led to propose a paracrine model of tumourigenesis, whereby oncogenic SOX2+ stem cells give rise to senescent clusters that induce tumour formation in a cell non‐autonomous manner [167, 168. More recently, a mechanism for this paracrine model has been postulated Figure 3. Gene profiling has revealed that mouse and human clusters are molecularly analogous and share a signature of senescence with activated SASP [48, 163. In agreement, cluster cells are sensitive to several senolytic agents [48, 95. Providing functional relevance, the attenuation of the senescence/SASP response in mice has been shown to result in a significant reduction in the tumour‐inducing potential of the cluster cells [48].

Together, this research has clearly demonstrated the presence of senescent cells in mouse and human ACP. A model is starting to emerge in which senescent cluster cells through the SASP play a critical role in tumour initiation in mouse ACP and tumour invasion in human ACP.

### Low‐grade gliomas

Low‐grade gliomas (LGGs) are a diverse group of benign brain tumours (WHO grade I and II). Symptoms are variable and largely attributable to mass effect from invasion into surrounding parenchyma (such as seizures, headache, cognitive or behavioural changes). LGGs are characterised by slow growth without invasive properties and generally low Ki‐67 proliferative index. Clinical management includes surgical resection if possible, radiation, chemotherapy and specific targeted therapies [169, 170, 171.

Senescence has been more thoroughly investigated in paediatric than adult LGG. Pilocytic astrocytoma (PA; WHO grade I) is the most prevalent paediatric LGG and the most frequent paediatric brain tumour in children. Constitutive activation of MAPK pathway, by genetic mutations, is detectable in nearly all cases [172], which leads to oncogene‐induced senescence, as shown by β‐galactosidase activity and induction of p16^INK4a^ expression in up to 90% of primary PA samples [173]. SASP factors (e.g. IL1B and IL6) are found to be upregulated in primary human tumours as well as in a PA mouse model [174]. SASP expression in PA tumours is associated with favourable prognosis whereas anti‐inflammatory treatment with dexamethasone inhibits the SASP and induces regrowth of senescent cells. These results highlight the importance of paracrine propagation and maintenance of senescence in paediatric LGG. Of relevance, senescent PA cells can be ablated using senolytics (i.e. ABT‐263 and ABT‐737), paving the way to a new type of treatment for these patients.

Homozygous deletion of *CDKN2A* (encoding p16^INK4a^) can be observed with low frequency in paediatric LGG [175], but is more common in higher‐grade tumours, such as pleomorphic xanthoastrocytoma and anaplastic astrocytoma with piloid features, suggesting that it probably acts as a second oncogenic hit, promoting senescence escape and facilitating transformation into high‐grade glioma [176, 177. Secondary alterations involving homozygous or hemizygous losses of *CDKN2A* and *TP53* are more characteristic in adult LGG. Increased survival has been associated with absence of mutations in *CDKN2A* and *TP53*, suggesting that senescence escape may promote tumour progression [178, 179.

Together, this research area has highlighted the presence of a large number of senescent cells in LGG. These cells through the SASP seem to play a critical role in tumour control, preventing the progression of the tumour to a more aggressive cancer. However, senescent cells in LGG are susceptible to a second oncogenic hit, promoting senescence escape and tumour progression.

### Glioblastoma multiforme

Glioblastoma multiforme (GBM; WHO grade IV) is one of the most common and aggressive primary brain tumours accounting for 60% of brain tumours in adults. They are highly infiltrative and have an average survival of less than 25% after two years due to the high recurrence rate [180]. GBM tumours contain heterogenous populations of cells characterised by various different genetic aberrations with a tendency to occur in any location in the brain. GBM tumours commonly show inhibition of the p53 and RB signalling pathways, or activation of RAS, PI3K and receptor tyrosine kinase pathways. Current standard of care therapy consists of surgical resection, adjuvant chemo/radiotherapy and administration of Temozolomide (TMZ) [181]. Even with this radical treatment regime, progression and recurrence rates are high and no other chemical treatments have shown great promise.

Evidence of therapy‐induced senescence in GBM has been shown following TMZ treatment and radiotherapy [182, 183. Culture of GBM cell lines in the presence of TMZ induces senescence through a DNA damage response pathway and expression of p21^Cip1^. Subsequently, the NF‐κB pathway is activated, accompanied by the production of the SASP components IL6 and IL8 [183, 184. Confirming the in vitro data, orthotopic transplantation of GBM cell lines into immunodeficient mice followed by oral administration of TMZ, leads to a senescence response evidenced by p21^Cip1^ expression and NF‐κB pathway activation in the tumour.

It is thought that radiotherapy in GBM leads to increased recurrence rates due to the induction of a tumour‐promoting microenvironment [182, 185. The DNA damage caused by irradiation results in the induction of senescence and SASP in both tumour cells and/or non‐tumour cells in the microenvironment, which as previously discussed can be pro‐tumourigenic and lead to recurrence [7, 15, 52. It has been shown that irradiation of GBM primary cells results in cellular senescence and SASP in vitro, as shown by morphological cellular changes, positive SA‐βgal staining, cell‐cycle arrest and p21^Cip1^ expression [51]. Upon irradiation, SASP factors such as IL6, IL1A and IL1B are induced and the NfκB pathway is activated. Furthermore, co‐injection of irradiated, senescent primary GBM cells with non‐irradiated GBM cells results in larger more aggressive tumours compared with the injection of only non‐irradiated cells in xenograft mouse models [188].

A recent study has demonstrated that GBM cell lines can be driven into senescence, by either TMZ treatment or irradiation, to subsequently be selectively ablated with Navitoclax (ABT‐263) as a senolytic [189]. Since the induction of senescence and SASP, caused by TMZ treatment or radiotherapy, can cause GBM recurrence, i.e. by creating a pro‐tumourigenic microenvironment favouring senescent escape or bypass, the ablation of GBM senescent cells could be expected to reduce tumour relapse and improve survival of the patients.

### Medulloblastoma

Medulloblastoma (MB; WHO grade IV)) is an embryonal tumour of the cerebellum originating from different neuronal progenitor cell populations. MB most commonly affects children and young adults with an average age of diagnosis of 6–8 years. MB is the most common high‐grade paediatric brain tumour. Gene expression analysis has subdivided this tumour into four major subgroups: WNT, SHH, Group 3 and Group 4 (Groups 3 and 4 have recently been subdivided into eight different types) [190, 191. These groups differ not only in their gene expression but also their methylation patterns, histology, clinical characteristics, metastatic potential, incidence and rate of recurrence. Despite the extensive clinical treatment stratification, outcomes of therapy can still be poor due to recurrence [192].

An initial study using a mouse model of SHH (sonic hedgehog) MB has identified p16^INK4a^ and p21^Cip1^ expressing senescent cells in the pre‐neoplastic MB lesions [46]. These senescent cells are not detectable in advanced tumours, which are characterised by the presence of spontaneous *p53* mutations, suggesting that senescence escape underlies tumour progression. This research has also shown that human SHH MB samples exhibit *CDKN2A* (encoding p16^INK4a^) promoter methylation, supporting a senescence evasion mechanism. Additional evidence of senescence has been proposed from in vitro studies using the cell lines DAOY and ONS‐76 [193]. Knockdown of citron kinase protein (CITK), which is required for normal proliferation and survival of neural progenitors, induces senescence and apoptosis via p53. In an MB mouse model, CITK deletion results in decreased tumour growth and increased overall survival, which is associated with increased expression of senescence markers such as p21^Cip1^, p27^Kip1^ and p16^INK4a^ in the tumours. There is a need to understand better the role of senescence in MB, not only as a tumour‐suppressive mechanism, but also the contribution of senescent cell‐mediated paracrine signalling to tumourigenesis.

### Diffuse Midline Glioma

Diffuse Midline Glioma (DMG) represents an incurable Grade IV group of paediatric tumours and accounts for 10% of all brain tumours in children. Found in the brainstem and midline structures including the thalamus, DMG are characterised by carrying mutations in histone‐encoding genes, i.e. histone H3 gene *H3F3A* (encoding H3.3) or in the related *HIST1H3B* (encoding H3.1) gene, often associated with loss of *TP53* [194]. Expression of senescence markers, such as p16^INK4a^, is very low in this tumour type, and this is probably due to the oncogenic driver mutations’ ability to represses the *CDKN2A* locus [195]. In contrast to the tumour cells, p16^INK4a^+ve cells are often found in the tumour microenvironment (up to 80% of tumours [196], suggesting that these potentially senescent cells could have a role in tumourigenesis and/or treatment resistance.

Conventional clinical management by radiotherapy or new targeted therapies could be used to trigger TIS in DMG tumours, as suggested by in vitro studies on patient‐derived DMG cell lines. The combination of radiation and the mTOR inhibitor AZD2014 has been shown to result in a strong synergistic antitumour activity preclinically [197], suggesting that the use of senolytics or SASP modulators could be of therapeutic relevance. Likewise, a recent study has proposed a new model where senescence is induced in DMG tumour cells by inhibition of BMI1. In vivo, the clearance of these treatment‐induced senescent cells with ABT‐263 attenuates tumour growth and prolongs animal survival [198].

## CONCLUDING REMARKS AND PERSPECTIVES

There is sufficient evidence to support the idea that senescent cells play a critical role in the pathogenesis of neurodegenerative conditions and brain tumours. The ablation experiments using genetic and chemical approaches have fuelled the interest in anti‐senescence therapies as potential treatments against these pathologies. However, several questions still remain that should be addressed to support further the development of senotherapies.

The function of senescent cells is highly context‐dependent. This has been better demonstrated in the cancer field, whereby senescent cells can be either anti‐ or pro‐ tumourigenic. This raises the possibility that senescent cells may also elicit beneficial or detrimental functions not just in cancer but also in other disease contexts. For instance, although a body of research has demonstrated the beneficial effects of ablating senescent cells with senolytics (e.g. by clearing organ‐resident senescent macrophages), a recent study using a new mouse model has revealed that the genetic ablation of vascular endothelial senescent cells in liver sinusoids, which express high levels of p16^INK4a^, results in premature death due to hepatic dysfunction [199]. This study highlights that the balance between the beneficial and detrimental functions of senescence must be thoroughly understood.

The ablation experiments in neurodegeneration mouse models suggest that senescent cells are not just bystanders, but they contribute to disease progression and cognitive loss. It will be interesting to assess whether such a role is preserved in humans. Another cerebral disease highly associated with old age is ischaemia/stroke and aneurysms [200]. During the acute phase of ischaemia in humans, pathogenic processes such as neuroinflammation (cytokines and chemokines) and oxidative stress have been shown to be upregulated. Furthermore, aged murine models have demonstrated a higher inflammatory response during the acute phase of ischaemia, which results in increased cerebral injury compared to young animals [200]. It will be important to define whether senotherapies are only able to prevent disease progression, or in addition, senolytics can improve cognitive decline and restore brain function in patients with advanced disease. These questions can be addressed in human trials, as those already running to test the efficacy of senotherapies against other human conditions.

Senescence is postulated to be a cell autonomous barrier against cancer that maintains potential cancer‐initiating cells in a benign, non‐proliferative state. Only through senescence bypass or senescence escape, caused by genetic or epigenetic alterations, can those benign lesion progress to give rise to malignant tumours. This model of cancer is supported mostly by in vitro studies and the fact that senescent cells are usually abundant in benign tumours while rare in malignant cancers [39, 40, 41, 43. Further studies using mouse models capable of genetic tracing the fate of senescent cells may clarify whether this model is universal and provide mechanistic insights. This is particularly important in view of data suggesting that senescent cells through paracrine signalling can, not only promote tumour progression to malignancy and metastasis, but also initiate tumour formation in a cell non‐autonomous manner [37].

One of the main problems with current anti‐cancer therapies is tumour recurrence. It is thought that senescent cells within the tumour bed are therapy‐resistant and will eventually re‐enter the cell cycle and give rise to a relapsed tumour. It has been shown that passing through a senescent state, even if transiently, can bestow features of stemness upon tumour cells making them more aggressive and malignant [12]. Therefore, there is a strong rationale to use senotherapies as adjuvant treatments to eliminate senescent cells prior to tumour recurrence. This is a promising approach, whereby current effective senescence‐inducing treatments, such as cytostatic chemotherapy, radiotherapy or specific targeted therapies, could be combined with senolytics in order to ablate the senescent cells prior to senescence escape and progression to recurrence. Preclinical research using suitable models of brain tumours will facilitate the development of clinical trials to test these combination therapies.

Despite the clear advantages of selectively eliminating senescent cells in mouse models in a variety of different human conditions, challenges remain to be addressed before senotherapies can be safely applied in clinical practice. Possibly, the most important one is to fully understand the mechanism involved in ‘good’ vs ‘bad’ senescence (i.e. beneficial vs detrimental effects). Such understanding requires a better characterisation of the different senescent cell populations within different organs in both normal physiology and disease contexts (e.g. specific diseases, ageing or cancer). This knowledge will provide a rationale for the use of senotherapies against certain human conditions and inform on the potential side effects, senolytic dosing regime, length of treatment and other important factors when designing clinical trials. Single cell profiling approaches and mouse models specifically designed to study senescence in vivo are ideal strategies to further our knowledge on the heterogeneity of senescent cells and reveal their functions. An additional problem is that there are very few drugs with proven senolytic activity and, in most cases, the mechanisms underlying such function remain poorly elucidated. There is an urgent need to discover novel senolytics and characterise their mechanisms of action through well‐designed senolytic screens.

We wonder whether Hayflick thought that his initial observations would ever become the catalyst that fuelled a vast research field, which potentially could improve clinical outcomes for the most relevant human diseases or even prolong a healthy life span. Despite current limitations and unknowns, it is difficult to not be affected by an encouraging optimism towards the translational potential of anti‐senescence therapies against brain pathologies and cancer. Future research will reveal key mechanistic insights into how senescent cells contribute to human disease paving the path to novel anti‐senescence treatments.

## ETHICS STATEMENT

Ethics approval was not required since this paper does not concern animal experimentation or the use of human volunteers.

## CONFLICT OF INTEREST

G.C. and R.G. have no conflicts of interest to declare. J.P.M.‐B. is an executive editor of *Neuropathology and Applied Neurobiology*. The Editors of *Neuropathology and Applied Neurobiology* are committed to peer‐review integrity and upholding the highest standards of review. As such, this article was peer‐reviewed by independent, anonymous expert referees and J.P.M.‐B. had no role in either the editorial decision or the handling of the paper.

## AUTHOR CONTRIBUTIONS

This paper was written, reviewed and approved by all three authors.

### Peer Review

The peer review history for this article is available at https://publons.com/publon/10.1111/nan.12689.

## Data Availability

Data sharing not applicable to this article as no datasets were generated or analysed during this study.
